# Key Competencies for Effective New Nurse Preceptorship: A Scoping Review of Essential Skills and Knowledge

**DOI:** 10.1155/jonm/4509979

**Published:** 2025-09-12

**Authors:** Qi Wang, Lamei Liu, Qin Zeng, Shaoyu Su, Shujing Gao

**Affiliations:** ^1^Department of Pediatric Respiratory and Immunology Nursing, West China Second University Hospital, Sichuan University, Chengdu, China; ^2^Key Laboratory of Birth Defects and Related Diseases of Women and Children (Sichuan University), Ministry of Education, Chengdu, China; ^3^Department of Pediatrics Nursing, West China Second University Hospital, Sichuan University, Chengdu, China

**Keywords:** competency, new nurses, nurse preceptors, nursing education, preceptorship

## Abstract

**Background:** The global nursing shortage necessitates rapid integration of new nurses into practice. However, the transition from student to professional remains challenging, with high first-year turnover rates. While preceptorship is vital for facilitating this transition, comprehensive competency frameworks for nurse preceptors are lacking.

**Aim:** This study aims to review and define key competencies of registered nurse preceptors, focusing on essential skills and developmental strategies to build a competency framework.

**Design:** A scoping review following established guidelines was conducted.

**Methods:** Systematic searches were performed in PubMed, Embase, Web of Science, and Cochrane Library from January 2012 to September 2022. The inclusion of studies was based on a set of defined eligibility criteria, and the results were synthesized into categories regarding the competencies of preceptors and methods for assessing them.

**Results:** A total of 48 studies were included in the final analysis. The competencies required for new nurse preceptors were categorized into four primary areas: (1) role model attributes, (2) teaching abilities, (3) motivational capacities, and (4) personal attribute competency. The review highlighted gaps in preceptor training, with many preceptors lacking formal education or institutional support. Additionally, the review found that the selection of preceptors and new nurses should be a mutual process, and preceptor competencies are closely linked to the effectiveness of induction training.

**Conclusions:** Healthcare institutions should prioritize the development of tailored preceptor training programs to ensure high-quality preceptorship for new nurses. Future research should focus on creating standardized frameworks for preceptors' competencies and evaluating the impact of specific training interventions to improve the effectiveness of preceptorship programs. By investing in preceptor education and fostering supportive environments, healthcare organizations can enhance nurse retention and contribute to the sustainability of the nursing workforce.

## 1. Introduction

A new nurse is someone who has completed their nursing education and has entered the nursing profession in the first year of employment [1]. The first 3 months of employment following graduation is the most stressful time in a nurse's career, and previous research has reported that a large percentage of new nurses may leave the profession in the first year [2]. According to a report published by the World Health Organization (WHO) in 2020, there is a global nursing shortage of 5.9 million. There is a need for action to improve new nurse retention.

New nurses face a host of challenges that impact their successful transition to practice. There may be fluctuations from the excitement of starting a career to negative emotions such as uncertainty, self-doubt, anxiety, and fear [3, 4]. With a stressful and high-work environment, coupled with a lack of confidence among new nurses, conflicting roles, unrealistic expectations of staff, lack of support, and strained relationships within their nursing colleagues even isolation or bullying, all these changes can lead to the loss of new nurse [5]. It is the responsibility of all workplaces to help new nurses navigate this period.

In China, nursing education typically involves a three- to 4-year program, which includes both theoretical coursework and clinical practice. Students are required to complete a minimum of 8–12 months of clinical practice in various areas, such as internal medicine, surgery, pediatrics, and obstetrics [6]. A graduate nurse often undergoes a structured preceptorship program, where they are paired with experienced new nurse preceptors to facilitate their transition into professional practice [7]. In China, newly graduated nurses typically do not obtain a nursing qualification certificate immediately upon graduation. Instead, they work as probationary nurses for the first 6 months, during which they engage in clinical practice under the guidance and supervision of preceptors before becoming formally registered [8]. These new nurse preceptors are usually selected based on their clinical expertise, teaching skills, and leadership qualities [9]. Additionally, there are training programs for nurse preceptors, which focus on teaching strategies, communication skills, and professional ethics [10]. However, given the large number of nursing schools and hospitals in China, substantial differences exist in educational philosophies and teaching approaches, leading to inconsistencies in the quality and standardization of preceptorship practices.

In the context of nursing, a “role” refers to the set of responsibilities, behaviors, and expectations associated with a specific position or function within the healthcare system [11]. Nurse preceptors, who are experienced registered nurses, play a critical role in guiding and supporting nurses at various stages of their professional development, including but not limited to newly graduated nurses during their transition from education to practice [12], Although this review highlights the role of preceptors in new nurse orientation and support, the competencies required for effective preceptorship are generally applicable for mentoring and supervising nurses across different levels of experience. To facilitate this transition, structured initiatives known as “programs” are designed to promote the professional development and integration of new nurses into the workforce [13]. Among these, preceptorship programs are formalized training frameworks that pair new nurses with experienced preceptors, providing preceptorship, skill development, and support during their initial employment phase [14].

A preceptor is described as a “specialized tutor who gives practical training to the student in the practice setting” [15]. It may be defined as a formal arrangement within a clinical setting between a practicing health professional (the preceptor) and a learner, such as a graduate nurse, student, or even an experienced nurse requiring guidance (the preceptee) [16, 17]. This trusting relationship aims to provide personal, professional, and career support and underlies the competencies required for effective preceptorship at various nurse experience levels.

However, being an effective preceptor for new nurses does not only require extensive clinical experience, but also a set of competencies related to teaching. Competencies are the knowledge, skills, and attitudes that individuals need to be able to perform their role effectively [16]. Currently, many different perspectives and frameworks exist regarding the competency of new nurse preceptors [16]. These frameworks typically include the following areas: teaching strategies, communication skills, feedback methods, evaluation criteria, and professional ethics [18]. However, there is a degree of inconsistency or overlap among these frameworks, and there is not a uniform or standardized definition or measurement [16]. In addition, these frameworks do not adequately consider the impact that factors such as different countries or regions, different cultural or linguistic backgrounds, and different areas or levels of expertise may have on the competency requirements for new nurse preceptors [18]. In the past, the focus on preceptors has been more for new nurse orientation and less on the role of the preceptor competence, organizational skills, the ability to guide, and direct others [19].

## 2. Methods

### 2.1. Aims

To conduct a scoping review of registered nurse competencies for new nurse mentoring/preceptorship and develop a competency framework.

### 2.2. Design

In this paper, the JBI scoping review method and the PRISMA-ScR report Guide are used for scoping review [20, 21]. We asked structured questions based on the background: (1) What definitions or descriptions of competency of new nurse preceptors are available in the literature? (2) What are the methods for assessing the competency of new nurse preceptors? (3) What are the factors that influence the competency of new nurse preceptors?

### 2.3. Eligibility Criteria

The inclusion and exclusion criteria were developed based on the objectives and research questions of this scoping review, as well as established methodological guidance for conducting scoping reviews. The criteria are summarized in Table 1.

### 2.4. Search Strategy

We conducted systematic searches in four databases: PubMed, Embase, Web of Science, and Cochrane Library. The search period is from January 2012 to September 2022. We used keywords and subject headings related to construct search statements; the snowball method was also used to search literature from other sources. The keywords were identified through an initial exploratory search and refined by the research team based on relevance to the review objectives. Both free-text terms and controlled vocabulary (e.g., MeSH) were used to ensure comprehensive coverage.  Table 2 presents the PubMed search strategy.

### 2.5. Study Selection

We use EndNote 20.0 software to manage the retrieved literature and remove duplicates. Two authors then screened the titles and abstracts based on inclusion exclusion criteria and resolved discrepancies. After title and abstract screening, the two authors again screened the full text based on the same inclusion and exclusion criteria and resolved discrepancies.

### 2.6. Data Charting process

Data that met the predefined inclusion criteria (including studies on preceptoring competencies for nurses at various experience levels) were extracted independently by two reviewers using a standardized form. The extraction process followed the Joanna Briggs Institute Reviewer's Manual (2020). Extracted data included the following: (1) author and year of publication; (2) country of origin; (3) study design; (4) journal; (5) research objectives; (6) population characteristics; (7) clinical setting; (8) key findings related to preceptor competencies; (9) theoretical frameworks; and (10) assessment tools used. Discrepancies were resolved through discussion.

## 3. Results

### 3.1. Characteristics of the Included Studies

**Figure 1:**
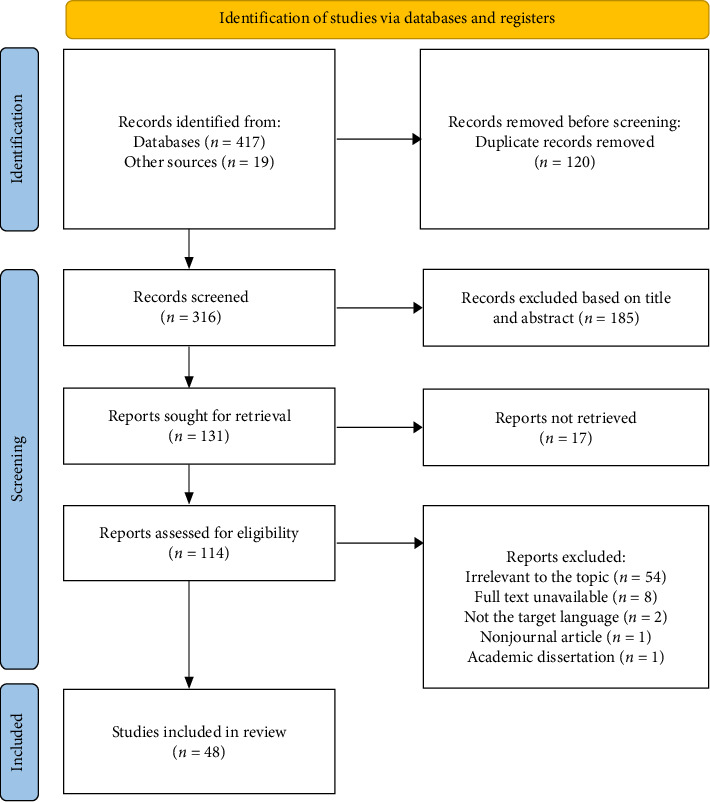
PRISMA 2020 flow diagram (adapted from Page et al. [81]).

A total of 48 studies met the inclusion criteria (Figure 1). The studies focused on six key themes: preceptorship (*n* = 7), preceptorship programs (*n* = 9), preceptor competence (*n* = 10), assessment (*n* = 11), new nurses' perceptions (*n* = 5), and preceptors' perceptions (*n* = 6).

### 3.2. Description of the Included Studies

The research design included 25 quantitative studies, 16 qualitative studies, 8 mixed methods, and 3 quasiexperimental designs (Table 3). These three types of quasiexperimental studies mainly discuss the specific characteristics of preceptors, the influence of cultural competence intervention on preceptors, and the influence of educational intervention on preceptors' training of new nurses' clinical competence (Table 4). Among the published countries, the US published the most in the last 10 years (10/48), followed by China (7/48) and Finland (5/48). It is worth noting that the Finnish studies were all published in the last 5 years.

### 3.3. Nurse Preceptor Competencies

Based on the included studies, several key competencies for nurse preceptors were identified. These include the following:

#### 3.3.1. Role Model

This category encompassed professional and personal attributes such as leadership, self-sacrifice, commitment, and the ability to serve as a positive example for new nurses. Studies reported that preceptors who consistently demonstrated ethical standards, professional behavior, and a commitment to continuous improvement positively influenced the practice transition of new nurses [1, 41, 63].

#### 3.3.2. Teaching Competency

Teaching competence combined pedagogical skills with clinical expertise. Effective preceptors were able to plan and adapt learning activities, provide constructive and timely feedback, facilitate clinical judgment and critical thinking, and apply evidence-based, competency-oriented curricula and assessment tools [25, 31, 46, 57]. Structured preparation before and during preceptorship, as well as flexibility to accommodate diverse learner needs, was repeatedly highlighted [35, 61].

#### 3.3.3. Motivation Competency

Motivation competence referred to a preceptor's dedication to the role and to developing the future nursing workforce. Factors that enhanced motivation included perceived benefits, recognition, rewards, and organizational as well as peer support [22, 30, 62, 63]. Workload management and clear role expectations were identified as critical to maintaining engagement and preventing burnout [34, 36, 56].

#### 3.3.4. Personal Attribute Competency

Key attributes for successful preceptorship encompassed interpersonal and communication skills, empathy, cultural competence, adaptability, and patience, while reflective practice was identified as a critical skill essential for effective mentoring [39, 49, 66]. These attributes and skills enabled preceptors to build strong relationships, support diverse learners, and navigate challenges in the clinical environment [42, 56].

Overall, this four-category competency framework integrated clinical proficiency, educational capability, motivational drivers, and relational attributes, reflecting a comprehensive approach to supporting the successful transition of new nurses into professional practice. Table 4 provides a detailed mapping of these competencies to the specific studies in which they were reported.  Table 5 presents the nurse preceptor competencies framework.

### 3.4. Description of the Theoretical Framework of the Included Studies

A total of 19 of the 48 included studies explicitly adopted a theoretical or conceptual frameworks to guide their research. Among these, three frameworks appeared most frequently: Kolb and Shugart's [67] Experiential Learning Theory, Benner et al.'s (1982) [68] Novice-to-Expert Theory, and Laschinger et al.'s [69] Model of Structural Determinants of Behaviour in Organisations. Kolb's theory emphasizes the cyclical process of learning from experience, which was applied in Oikarainen et al. [37] and Tuomikoski et al. [43] to structure preceptor training interventions and evaluate learning outcomes. Benner's theory focuses on the progressive acquisition of clinical skills and professional judgment was used in studies such as Rodrigues and Witt [46] and Lin et al. [39] to frame the assessment of preceptors' clinical and teaching abilities. Kanter's model focuses on how organizational resources and leadership structures affect staff behavior, which was adopted in studies like Gholizadeh et al. [63] to analyze institutional support for preceptors.

Other frameworks were also presented, including Boychuk Dutcher's (2009) [70] theory of transition shock, which helped elucidate the adaptation challenges new nurses face, as discussed in Martínez-Linares et al. [60]; Colaizzi's (2003) phenomenological framework, applied for qualitative analysis of preceptors' experiences in Panzavecchia and Pearce [56]; and Sonthisombat's (2008) competency model, used by Hsu et al. [57] to assess cultural competence. Although these frameworks differ in focus—ranging from individual psychological adjustment to qualitative experience analysis and cultural competency—they all serve to theoretically underpin preceptor competency models, guiding both research and practical approaches to enhance preceptorship effectiveness. It should be noted that these frameworks are not mandated universally but are selected based on study objectives and contextual relevance, offering valuable perspectives that inform model development and application.

## 4. Discussion

This scoping review successfully achieved its aim of identifying key competencies for nurse preceptors to support the transition of new nurses into professional practice. A total of 48 studies were included in the review, providing a comprehensive and diverse evidence base to inform the development of competency frameworks and practical recommendations.

### 4.1. Preceptorship

Preceptorship has been proven to be effective in reducing the turnover rate of new nurses and improving their satisfaction [15, 16]. Preceptorship programs serve as best practices for new nurse transition practice programs [35, 63, 71]. The form and period of implementation vary from countries and institutions, and also may depend on the level of policy and departmental support for them and the level of expectations for new nurses. The time span can vary from days to weeks even years and is entirely up to the training provider [27, 55, 72]. What is conducted also varies; some projects recommend that preceptorship programs be evidence-based and structured, and some suggest that it would be best to work with institutions to train nurses with preceptorship qualifications in advance [29, 58, 73]. Also, a successful preceptorship is a two-way process, requiring the combined efforts of both preceptors and trainees [44].

### 4.2. Nurse Preceptor Competencies

#### 4.2.1. Role Model Competency

Our review found that nurse preceptors acted as role models by demonstrating professional commitment, ethical standards, and leadership, thereby influencing new nurses through example. This aligns with previous research, indicating that effective preceptors transmit professional values and behaviors to preceptees, fostering professional identity and clinical competence [1, 41, 63, 64]. Literature also notes that role modeling extends beyond technical skills to encompass professional attitudes, self-sacrifice, and continuous improvement [59]. While some authors debate whether self-sacrifice is essential [62], our findings suggest that role modeling is most effective when preceptors are visibly committed to both patient care and the development of new nurses. Organizational support, such as recognition and workload adjustment, may further enhance preceptors' ability to serve as effective role models.

#### 4.2.2. Teaching Competency

Teaching competency encompassed pedagogical skills, clinical expertise, evaluation, feedback, and the ability to adapt instruction to diverse learners. The literature supports our finding that teaching preparation is fundamental to preceptorship [24, 31, 39, 45–48, 57, 59]. Structured training in areas such as conflict resolution, adult learning principles, communication, feedback delivery, and critical thinking has been shown to improve preceptor performance [23, 33, 44, 46, 60]. Our results also indicated that effective teaching requires not only knowledge transfer but also facilitation of clinical judgment and evidence-based decision-making. Assessment mechanisms, including self-evaluation and feedback from new nurses, remain essential for identifying areas of improvement [23, 28, 60]. Feedback loops—whether formal or informal—have been found to increase new nurse satisfaction [42, 53, 54]. Therefore, training programs should integrate both teaching skills and mechanisms for ongoing evaluation.

#### 4.2.3. Motivation Competency

Motivation competency reflected preceptors' dedication to their role, shaped by both intrinsic factors (e.g., professional pride, satisfaction) and extrinsic supports (e.g., recognition, managerial backing, organizational resources). Our findings align with prior research, showing that motivation increases when preceptors perceive benefits, receive recognition, and have clear role expectations [15, 22, 30, 34, 36, 56, 62, 63]. Conversely, excessive workload, unclear expectations, and lack of support can diminish motivation and affect retention. Organizational interventions, such as workload management and access to peer support networks, may sustain motivation. The transition from experienced nurse to preceptor also requires an adjustment in professional identity, which may be supported by preceptorship from seasoned preceptors [55, 56].

#### 4.2.4. Personal Attribute Competency

Attributes such as communication skills, empathy, cultural competence, and adaptability were identified as key facilitators of successful mentoring relationships. Reflective practice, as a distinct skill, was also found to play a pivotal role in enabling preceptors to manage stress, address diverse learner needs, and promote mutual growth. Our findings are supported by studies emphasizing the importance of emotional intelligence, interpersonal skills, and reflective practice in effective preceptorship [39, 49, 66]. While confidence is recognized as an important attribute, some preceptors expressed uncertainty when guiding nurses with learning challenges [48–51]. Tailored training and experiential learning could enhance preceptors' confidence and reflective practice skills in such scenarios.

### 4.3. Assessment

Several scales are used to assess preceptor competencies, the main components including skill acquisition, identification of developmental directions, preceptor orientation competency assessment, subjective competency assessment, and competency assessment. Self-evaluation or feedback from new nurses is the main test method. The main findings from the assessment fell into several categories: preceptor unpreparedness, overexpectation, and poor adaptation of the preceptor to the role. These findings also confirm the issue that preceptors need training and support [23, 28, 60].

### 4.4. Perceptions of Preceptors

New nurses consider that preceptors are critical to their transition from student to registered nurse and can shorten this transition period [71, 74]. However, preceptors appear less confident when dealing with new nurses with learning disabilities, and they are not prepared to deal with such new nurses [54, 56, 59, 75].

Experienced nurses need to make a role change when faced with a new preceptor role. They need to pass on their knowledge and skills or experience to new nurses, which requires skills and assessment. It seems that educators and facilitators are not yet aware of the complexity of the relationship and the difficulty of the transition. Potentially influencing factors include nursing position readjustments, interunit transfers, frequent staff rotations, and the workload associated with the preceptor role [76, 77]. Kanter's model of structural determination of behavior states that it takes opportunity and support to develop fidelity to one's role and it might be useful to ask our preceptors what areas they need training or improvement in [15].

### 4.5. Synergistic Theoretical Perspectives in Preceptorship Research

The included studies span multiple countries and employ diverse research designs, reflecting the multidimensional and complex nature of preceptorship research. The United States, China, and Finland emerged as the primary contributing countries, with Finland showing a notable increase in publications in recent years. Among these studies, the most frequently adopted theoretical frameworks include Kolb's “Experiential Learning Theory”, Benner's “From Novice to Expert” model, and Kanter's “Structural Determinants of Behavior in Organizations” model.

These models offer complementary perspectives: Kolb emphasizes the cyclical process of learning and reflective observation, making it suitable for guiding individual learning and competency development [67]. Benner focuses on the progressive accumulation of skills and the enhancement of clinical judgment, highlighting the developmental trajectory from novice to expert [68]. However, it has limitations in addressing highly complex and dynamic clinical contexts, such as overlooking cognitive biases and situational factors [78]. Kanter emphasizes institutional resources and leadership support, underscoring the influence of organizational structures on behavior [69]. Yet, in rapidly changing environments, it struggles to fully account for individual autonomy and innovation, and further refinement is needed to enhance its applicability in fostering innovation and managing complexity.

Future research should explore the integration of multiple models, incorporating individual psychology, organizational support, and environmental dynamics to develop more comprehensive and resilient support strategies for nursing career development [79, 80]. Additionally, attention should be paid to the limitations of existing models, with efforts directed toward developing context-adaptive theoretical frameworks that can better address the rapid changes and diverse demands of clinical practice.

### 4.6. Implications for Practice

Across all four competency domains, targeted training and organizational support are necessary to ensure that preceptors are well-prepared for their role. Preceptor preparation programs should be evidence-based, competency-oriented, and aligned with the four domains identified in this review. Furthermore, establishing standardized criteria for preceptor selection—such as educational background, work experience, and demonstrated competencies—may improve the quality and consistency of preceptorship programs [35, 52].

## 5. Limitations

Despite the valuable insights gained from this scoping review, several limitations should be acknowledged. First, this review is limited by its language scope (English only), which may exclude relevant studies globally. Second, most included studies originate from high-income countries, restricting generalizability to low- and middle-income settings. Third, the heterogeneity of study designs and lack of standardized assessment tools may affect reliability. Additionally, the evolving nature of nursing preceptorship suggests ongoing updates are needed. Future research should address these limitations and evaluate training interventions across diverse contexts.

## 6. Conclusions

This scoping review highlights the critical role of nurse preceptors in supporting the professional growth of new nurses, particularly during their transition into the workforce. The competencies required for new nurse preceptors are multifaceted, encompassing role model attributes, teaching abilities, motivational capacities, and personal characteristics. These competencies are not only essential for fostering the development of new nurses but also directly impact the effectiveness of preceptorship programs. While the literature emphasizes the importance of preceptor training, the findings reveal a significant gap in the formal preparation and institutional support available for preceptors. Many preceptors lack adequate training in the necessary skills for precepting, leading to variations in the quality of preceptorship across institutions. Therefore, it is imperative for healthcare institutions to prioritize preceptor training programs, ensuring that they are evidence-based and tailored to meet the specific needs of preceptors and new nurses alike.

Moreover, the success of preceptorship programs depends on a mutual and collaborative approach between preceptors and new nurses, underpinned by clear communication, support, and continuous professional development. Future research should focus on developing standardized frameworks for preceptor competencies and evaluating the effectiveness of training programs to enhance the quality of preceptorship and improve the retention and job satisfaction of new nurses. By investing in preceptorship training and fostering supportive environments, healthcare institutions can help ensure a smoother transition for new nurses and contribute to the long-term sustainability of the nursing workforce.

## Figures and Tables

**Table 1:** Literature inclusion and exclusion criteria.

Inclusion criteria	Exclusion criteria
(1) English language articles published in peer-reviewed journals; (2) Articles related to the competency of nurse preceptors, including both new nurses and experienced nurses or preceptors; (3) The definition, description, framework, or model of preceptor competencies for preceptoring and supervising nurses at any level.	(1) Non-English article; (2) Non–peer-reviewed journal articles; (3) Articles that are not relevant or sufficiently detailed to the competency of new nurse or experienced nurses preceptors.

**Table 2:** PubMed search strategy.

Query	Search items
#1	(((preceptor[Title/Abstract])) OR (preceptorship[MeSH Terms])) OR (mentors[MeSH Terms])
#2	(education[Title/Abstract]) OR (“Education, Nursing”[Mesh])
#3	((((clinical competence[MeSH terms]) OR (professional competence[Title/Abstract])) OR (core competence[Title/Abstract])) OR (competence[Title/Abstract])) OR (skill[Title/Abstract])
#4	((((((((new^∗^ graduate nurse^∗^ [Title/Abstract]) OR (newly qualified nurse^∗^[Title/Abstract])) OR (newly registered nurse^∗^[Title/Abstract])) OR (newly hired nurse^∗^[Title/Abstract])) OR (new nurse^∗^ [Title/Abstract])) OR (novice nurse^∗^[Title/Abstract])) OR (neophyte nurse^∗^[Title/Abstract])) OR (rookie nurse^∗^[Title/Abstract])) OR (advanced beginner^∗^[Title/Abstract])
	#1 AND #2 AND #3 AND #4

*Note:* The search period for this strategy was from January 2012 to September 2022.

**Table 3:** Basic characteristic of included studies.

Theme	Author and year of publication	Country	Method design	Journal
Preceptorship	Hong and Yoon 2021 [22]	Korea	Descriptive cross-sectional study	International Journal of Environmental Research and Public Health
	Van Patten and Bartone 2019 [17]	USA	Cross-sectional study	Nurse Education in Practice
	Zhang et al. 2019 [23]	China	Longitudinal, nonrandomized control study	Nurse Education in Practice
	McSharry and Lathlean 2017 [24]	Ireland	Qualitative study	Nurse Education Today
	Hu et al. 2015 [25]	China	Repeated-measures design	Journal of Continuing Education in Nursing
	Cooley and De Gagne 2016 [26]	USA	Qualitative study	Journal of Nursing Education
	Figueroa et al. 2013 [27]	USA	Mixed method	Journal of Continuing Education in Nursing

Preceptorship program	Taylor, Eost-Telling, and Ellerton 2019 [28]	UK	Mixed method	Journal of Clinical Nursing
	Sheppard-Law et al. 2018 [29]	Australia	Qualitative study	Contemporary Nurse
	Scott-Herring and Singh 2017 [30]	USA	Quality improvement project	Journal of Continuing Education in Nursing
	Mårtensson et al. 2016 [31]	Sweden	Qualitative study	Nurse Education in Practice
	Clipper and Cherry 2015 [32]	USA	Project	Journal of Continuing Education in Nursing
	Sharpnack, Moon, and Waite 2014 [33]	USA	Pilot project	Journal for Nurses in Professional Development
	Muir et al. 2013 [34]	UK	Mixed method	Nurse Education Today
	Marks-Maran et al. 2013 [35]	UK	Mixed method	Nurse Education Today
	Haggerty, Holloway, and Wilson 2012 [36]	New Zealand	Longitudinal study	Nursing Praxis in New Zealand

Competence	Oikarainen et al. 2022 [37]^∗^	Finland	Quasiexperimental study	Nurse Education Today
	Pohjamies, Haapa, et al. 2022 [38]	Finland	Cross-sectional study	Journal of Advanced Nursing
	Lin et al. 2021 [39]	China	Cross-sectional study	Nurse Education Today
	Tuomikoski et al. 2020 [40]^∗^	Finland	Quasiexperimental design	Scandinavian Journal of Caring Sciences
	Borimnejad et al. 2018 [41]	Iran	Qualitative study	Nurse Education in Practice
	L'Ecuyer, Hyde, and Shatto 2018 [42]	USA	Qualitative study	Journal of Continuing Education in Nursing
	Tuomikoski et al. 2018 [43]^∗^	Finland	Cross-sectional study	Nurse Education Today
	Bengtsson and Carlson 2015 [44]	Sweden	Qualitative study	BMC Nursing
	Knisely, Fulton, and Friesth 2015 [45]	India	Cross-sectional study	Journal of Professional Nursing
	Rodrigues and Witt 2013 [46]	Brazil	Descriptive study	Journal of Continuing Education in Nursing

Assessment	Sebach 2022 [47]	USA	Descriptive study	JNP—Journal for Nurse Practitioners
	Pohjamies, Mikkonen, et al. 2022 [48]	Finland	Instrument development	Nurse Education in Practice
	Mauri et al. 2020 [49]	Italy	Observational multicenter transversal study	Nursing Reports
	Chen et al. 2019 [50]	China	Cross-sectional study	Journal of Continuing Education in Nursing
	Wilburn, Jones, and Hamilton 2018 [51]	USA	Pre- and postintervention design	Journal for Nurses in Professional Development
	Tiew et al. 2017 [52]	Singapore	Pre- and postintervention design	Nurse Education Today
	Lee-Hsieh et al. 2016 [53]	China	Mixed method	Nurse Education Today
	Nielsen, Lasater, and Stock 2016 [54]	USA	Qualitative descriptive substudy	Nurse Education in Practice
	Bradley et al. 2015 [55]	USA	Methodological study	Journal for Nurses in Professional Development
	Panzavecchia and Pearce 2014 [56]	UK	Qualitative study	Nurse Education Today
	Hsu et al. 2014 [57]	China	Quantitative study	Contemporary Nurse

Perceptions of preceptors (new nurse)	Hansen 2021 [58]	South Africa	Cross-sectional study	Curationis
	L'Ecuyer 2019 [59]	USA	Cross-sectional study	Nurse Education in Practice
	Martínez-Linares et al. 2019 [60]	Spanish	Qualitative study	International Journal of Environmental Research and Public Health
	Quek et al. 2019 [61]	Singapore	Qualitative study	Nurse Education in Practice
	Moore and Cagle 2012 [1]	USA	Qualitative study	Journal of Continuing Education in Nursing

Perceptions of preceptors (preceptors)	Alhassan et al. 2022 [62]	Ghana	Cross-sectional study	Nurse Education Today
	Gholizadeh et al. 2022 [63]	Iran	Descriptive study	BMC Medical Education
	Macey, Green, and Jarden 2021 [64]	Australia	Cross-sectional mixed-method study	Nurse Education in Practice
	Omer, Suliman, and Moola 2016 [65]	Saudi Arabia	Descriptive and comparative study	Nurse Education in Practice
	Chang et al. 2015 [66]	China	Mixed method	Nurse Education Today
	Cloete and Jeggels 2014 [15]	South Africa	Descriptive study	Curationis

^∗^Findings on mentor training may also apply to new nurse preceptors.

**Table 4:** Overview of included studies.

Author and year	Purpose	Population	Setting	Key findings	Theoretical framework	Research scale	Nurse preceptor competency required
Hong and Yoon 2021	Investigated preceptors' experiences in educating new nurses; effect on Clinical Teaching Behavior (CTB) based on preceptor training participation	Preceptors (*n* = 180)		Preceptors need manager and colleague support; develop preceptor training programs		CTB Inventory (CTBI-22), Cronbach's *α* = 0.92	Managerial and Peer Support; Training Program Development

Van Patten and Bartone 2019	Identified factors enhancing positive nurse-residency experiences	Graduate nurses (*n* = 1078)	Various hospitals throughout the United States	Higher residency ratings linked to better preceptor and debriefing experiences; no significant stress reduction effects on preceptorship/debriefing-residency rating relationship; new nurses benefit from programs with mentorship, preceptors, and debriefing; debriefing sessions enhanced skills and confidence in first employment year	Roy Adaptation Model and Benner's Novice-to-Expert Theory	1. Demographic Information Survey 2. Evaluation of registered nurse Residency Survey	Mentorship and Debriefing Skills; New Nurse Support

Zhang et al. 2019	Assessed one-on-one mentorship program effectiveness in reducing nurse turnover in China	New graduate nurses; control group (*n* = 199) and experimental group (*n* = 239)	A tertiary general hospital located in the Hangzhou, Zhejiang Province of China	One-on-one mentorship program benefits new graduate nurse retention, especially in first year			One-on-One Mentoring

McSharry and Lathlean 2017	Explored clinical teaching and learning in preceptorship model at Irish acute care hospital	Students (*n* = 13) and preceptors (*n* = 13)	One hospital in Ireland in four clinical areas	Preceptors need educational preparation and support for pedagogical competencies; develop national competency-based standards			Educational Preparation and Pedagogical Competence

Hu et al. 2015	Developed 10-minute preceptor model for preceptor-new graduate nurse interaction in first 3 months; compared work stress, turnover intention, experience, and satisfaction between 10MP and TPM groups	New graduate nurses (*n* = 107)	A district teaching hospital with two branches in New Taipei City: Taiwanese hospitals	10-min preceptor model improves training outcomes and new graduate nurse professional development		1. Work stress questionnaire 2. Work experience scale 3. Satisfaction with the preceptors	Time-Efficient Preceptor Training Model

(Cooley and De Gagne 2016)	Explored novice nursing faculty experiences; examined facilitators and barriers to nurse educator competence; identified transformative learning experiences	Novice nursing faculty (*n* = 7)		Internship programs essential for novice nursing faculty competence development	Kolb's (1984) experiential learning theory		Internship Program Facilitation

Figueroa et al. 2013	Explored first-year turnover rates of new graduate RNs; perceptions of MSPM in transitioning RNs to practice	New graduate RNs (*n* = 108) and preceptors (*n* = 100)	A seven-hospital system in the southeast region of the United States	MSPM retains quality nursing workforce focused on patient safety by transitioning new graduate RNs to practice			Workforce Transition and Patient Safety Focus

Taylor, Eost-Telling, and Ellerton 2019	Reviewed and analysed preceptorship programmes in Northwest England NHS trusts; evaluated pedagogic rigour; suggested future design recommendations	NHS trusts (*n* = 41)	Online survey	Nursing staff shortage in England ongoing; recruitment and retention key; preceptorship improves retention of newly qualified staff			Recruitment, Retention, and Preceptorship

Sheppard-Law et al. 2018	Explored novice clinical nurse educator's learning and mentoring experiences	Clinical nurse educator (*n* = 19)	Two tertiary hospitals	Self-directed and mentoring programs transform novice educator practice; consolidate knowledge, skills, and confidence to educate less experienced nurses			Self-Directed Learning Facilitation and Mentoring Program Delivery

Scott-Herring and Singh 2017	Preceptorship-mentorship program increases preceptor and oriental satisfaction, confidence, and comfort; project determined effects of these outcomes	Newly registered nurse anesthetist preceptors (*n* = 12) and orientees (*n* = 3–5)	CRNA division in mid-Atlantic academic center; evidence-based preceptorship–mentorship program increased CRNA preceptor satisfaction and comfort	An evidence-based CRNA preceptorship–mentorship program significantly increased CRNA preceptor satisfaction and comfort		CRNA Preceptor Pre-Program Survey	Specialized Preceptorship and Mentorship (e.g., CRNA)

Mårtensson et al. 2016	Described preceptors' educational role experiences before and after university preceptor preparation course	Preceptors (*n* = 27)		Reflective learning in preceptor preparation course strengthens preceptors' view of educational role; helps manage and create preconditions for preceptorship	Self-directed learning (Knowles, 1975)		Reflective Learning and Educational Role Management

Clipper and Cherry 2015	Described implementation and evaluation of preceptor development program; effect on NGRN transition to practice	New graduate nurses (*n* = 138)	A seven-hospital system	Structured preceptor–training program improves NGRN transition to practice and first-year retention rates	Boychuk Duchscher's (2009) theory of transition shock	16-item, investigator-developed survey based on transition shock theory attributes (Boychuk Duchscher, 2009)	Structured Preceptor Training Implementation

Sharpnack, Moon, and Waite 2014	Guided preceptors as educators; fostered new nurse competencies; facilitated transition to professional practice	Preceptors (*n* = 19)	Six hospitals in northeast Ohio	Academic partnerships essential for transition through strategic immersion programs; nursing schools should educate preceptors, design immersion programs to reduce costs, and develop leadership competencies for safe transition to practice			Academic Partnership and Leadership Competency Development

Muir et al. 2013	To evaluate the preceptors' perception of the preceptorship programme	Preceptors (*n* = 9) and experienced nurses (*n* = 90)	One NHS Healthcare Trust in Southwest London, UK	Preceptors viewed preceptorship program and their role positively; time constraints with preceptees an issue; positive impact on preceptee, organization, and preceptor development			Positive Role Attitude and Time Management

Marks-Maran et al. 2013	Evaluated preceptorship programme for newly qualified nurses; determined preceptee engagement, impact, value, and sustainability from preceptees' perspectives	Newly-qualified nurses (*n* = 90)	One NHS Healthcare Trust in South West London, UK	Best practice: Appropriate preceptor selection, adequate education, and support for new graduate nurses; New Zealand nurses expected to use ABC approach for smoking cessation; not all nurses adequately prepared		The questionnaires were developed by the Ministry of Health (MoH) and piloted in 2004 across three Nursing Entry to Practice sites	Appropriate Preceptor Selection, Education, and New Graduate Support

Haggerty, Holloway, and Wilson 2012	Evaluation aimed to gather lessons from Nursing Entry to Practice programmes in New Zealand; identify new graduate and DHB success factors; share resources to support national best practice development	Nursing Entry to Practice programs (*n* = 21)		Recommended clearly defined preceptor selection process; flexible preceptor education programs to support new graduates in first year of practice; no increase in preceptor workloads	Fourth generation evaluation (FgE) (Guba & Lincoln, 1989)		Selection Process Clarity and Flexible Education Programs

Oikarainen et al. 2022	Evaluated effects of educational intervention on mentors' competence in mentoring diverse nursing students during clinical placement	Mentors; intervention group (*n* = 49), Control group (*n* = 62)	Two hospitals located in Finland	Study provided evidence on education to improve mentors' competence; helps diverse nursing students overcome clinical placement challenges; reinforcement strategies needed for long-term competence maintenance	Evidence-based clinical mentors' competence model	1. Mentors' Competence Instrument 2. Mentors' Cultural Competence Instrument	Mentor Competence Improvement and Long-term Reinforcement

Pohjamies, Haapa, et al. 2022	To identify distinct orientation competence profiles amongst nurse preceptors and explain the associated factors	RNs (*N* = 8279; *n* = 844 completed)	One Finnish university hospital	Preceptor selection based on orientation competence, not availability; preceptors need co-worker support to focus on orientation tasks; further orientation education needed to outline learning goals and assess performance; clinical experience, motivation, willingness, and prior education influence orientation competence	A Bachelor's level program representing European Qualification Framework level 6 (European Union, EQF levels 2021)	Self-administered, electronic Finnish version of Preceptors' Orientation Competence Instrument; Cronbach's *α* = 0.79–0.93	Orientation Competence and Peer Support

Lin et al. 2021	Compared pre-graduate students, newly graduated nurses, RNs, and nurse mentors in Taiwan on cultural competence levels; determined influencing factors	Pre-graduate nurses (*n* = 103), newly graduated nurses (*n* = 321), RNs (*n* = 101), and nurse mentors (*n* = 231)	Hospital A and hospitals B, C	Pregraduate students, newly graduated nurses, RNs, and nurse mentors have differing cultural competence levels; nurse mentors scored higher than newly graduated nurses; continuous improvement of cultural competence essential		Cultural Competence Scale for Pre-Graduate Students to Licensed Professionals	Cultural Competence and Continuous Improvement

(Tuomikoski et al. 2020)	Evaluated how educational intervention affects nurse mentors' competence in mentoring nursing students during clinical practice	Nurse mentors (*n* = 120)	One university hospital and two central hospitals in Finland	Internationally, nurse mentors not typically required to complete mentoring education; mentoring education increases competence; recommend all nurse degree programs include mentoring education		Mentor Competence instrument; Cronbach's *α* = 0.703–0.891	Mentoring Education Integration

Borimnejad et al. 2018	To explore the attributes of new nurse preceptors	Preceptors (*n* = 6)	A large pediatric teaching hospital in Northwest Iran	Preceptors are role models; self-sacrifice spirit transfers to new nurses and future preceptors; effective preceptorship requires specific professional and personal attributes	Heidegger's philosophy		Role Modeling and Professional/Personal Attributes

L'Ecuyer, Hyde, and Shatto 2018	Identified defining characteristics of preceptor competency after attending day-long preceptor academy	Preceptors (*n* = 553)	Online survey	Understanding role expectations benefits preceptors and nurse educators; preceptor training includes skills, knowledge, and attitudes; competence development relies on preparation courses, skill development, and role modeling; preceptor programs should cultivate continuous improvement			Understanding Role Expectations and Continuous Development

Tuomikoski et al. 2018	Described and explained nurse mentor competence in mentoring nursing students in clinical practice based on self-evaluation; identified different mentor profiles	Mentors (*n* = 3355)	Five university hospitals in Finland	Mentors have diverse support needs for building mentoring competence; healthcare organizations should provide education based on individual competence levels; encourage reflective discussion with students during clinical practice		Mentors Competence Instrument	Individualized Support and Reflective Discussion Encouragement

Bengtsson and Carlson 2015	Investigated and included preceptors' requests and educational needs in developing advanced continuous professional development course	Preceptors (*n* = 64)	Healthcare sector in south of Sweden	Vital preceptor preparation components: teaching and learning strategies, reflective and critical reasoning, communication models, preceptor role, preceptorship			Comprehensive Preceptor Preparation Components

Knisely, Fulton, and Friesth 2015	Explored and compared CNS student and preceptor perceptions of clinical teaching characteristics in CNS preceptors	Preceptors (*n* = 278); students (*n* = 78)	Online survey	Clinician preceptors instrumental in developing students' CNS practice competencies; 21 effective clinical teaching characteristics important for CNS preceptors			Effective Clinical Teaching Behaviors

Rodrigues and Witt 2013	Identified required competencies for preceptorship in Brazilian health care system	Preceptors in nursing (*n* = 9)	The Federal University of Rio Grande do Sul and the Health District Glória/Cruzeiro/Cristal	Preceptors committed to educating future health care professionals; emphasized pedagogical skill development and necessity of education before preceptorship			Commitment to Education and Pedagogical Skill Development

Sebach 2022	To assess the psychometric properties of the Academic Clinical Nurse Educator Skill Acquisition Tool (ACNESAT) with nurse practitioner clinical educators	Nurse practitioner (*n* = 137)	Online survey	ACNESAT assesses skill acquisition, identifies professional development needs, and guides individualized mentoring programs		ACNESAT. Cronbach's *α* = 0.972	Skill Acquisition Assessment and Individualized Mentoring Guidance

Pohjamies, Mikkonen, et al. 2022	Developed and psychometrically tested Preceptors' Orientation Competence Instrument to measure orientation competence of new employee preceptors	RNs (*n* = 844)	A Finnish university hospital	Preceptors' Orientation Competence Instrument demonstrated adequate psychometric properties; useful for self-evaluation of orientation competence of new employee preceptors; Cronbach's alpha 0.96 (range 0.79–0.98)	1. An integrative review about the orientation competence of nurses working as new employee preceptors (Voutilainen et al. 2019) 2. Mentor Competence Instrument (Mikkonen et al., 2020)	Preceptors Orientation Competence Instrument	Self-evaluation of Orientation Competence

(Mauri et al. 2020)	Created and validated midwifery preceptor's evaluation form for use by midwifery students	Students (*n* = 88)	University of Milan	Provide constructive feedback to students during and after clinical placements; feedback can be given anytime, maintaining privacy and promoting professional development		A midwifery preceptor's assessment questionnaire, alpha score of 0.97–0.85	Constructive Feedback Provision

Chen et al. 2019	Study conducted in two stages: collected information on preceptor training courses; conducted cross-sectional questionnaire survey	Preceptors (*n* = 350)	Preceptors from eight acute care hospitals in the greater Taipei region	Subjective Competency Scale (SCS) exhibited high validity and reliability; useful for future preceptors' subjective competency assessment and evaluation		SCS, Cronbach's *α* = 0.715–0.889	Subjective Competency Assessment

Wilburn, Jones, and Hamilton 2018	Identified preceptor confidence in evaluating preceptee performance during new graduate nurse orientation	Preceptors (*N* = 33; *n* = 15 completed)	Two urban medical centers and one community hospital in the southeastern United States	After standardized evaluation process for new graduate nurses, individual scores increased from 17–25 (preintervention) to 20–25 (postintervention); mid to high preceptor confidence		1. Norway Nurse Competence Scale 2. Revised Confidence Scale, Cronbach's *α* = 0.83	Confidence Development through Standardized Evaluation

Tiew et al. 2017	Developed and tested instrument to measure graduate-nurses' perceptions of structured mentorship program	Graduate nurses in second year residency program (*n* = 84)	Large metropolitan tertiary hospital	National University Hospital Mentorship Evaluation (NUH ME) measure valid and reliable; mentorship programs effective for recruitment and retention but resource intensive	Patricia Benner's Novice to Expert model (1984)	NUH ME, Cronbach's *α* = 0.92	Mentorship Program Effectiveness and Resource Management

Lee-Hsieh et al. 2016	Developed Clinical Teaching Behavior Inventory (CTBI) for nurse preceptors' self-evaluation and new graduate preceptee evaluation of clinical teaching behaviors; tested validity and reliability	Phase II: preceptors (*n* = 63) and new graduate (*n* = 24) Phase IV: 290 nurse preceptors (*n* = 290) and new graduate nurses (*n* = 260)	Five teaching hospitals	CTBI-23 valid and reliable for identifying preceptor clinical teaching behaviors as perceived by preceptors and new graduate preceptees; depicts clinical teaching behaviors of nurse preceptors in Taiwan; Cronbach's *α* 0.96 (*n* = 521)		CTBI-23	Identification and Measurement of Clinical Teaching Behaviors

Nielsen, Lasater, and Stock 2016	Gained insight about new clinical judgment assessment process from preceptors' lived experiences	Experienced preceptors (*n* = 7)	Urban medical center in the U.S.	Noticing, Interpreting, Responding, and Reflecting informed strategies; structured framework provided objective ways to evaluate and develop new graduate nurses' clinical judgment; useful for preparing students for transition to practice	The Tanner Model of Clinical Judgment (2006)		Framework-based Clinical Judgment Development

Bradley et al. 2015	Developed and tested Baptist Health Lexington Performance and Proficiency Assessment (PPA) for validity and reliability	Nurse experts (*n* = 12) Nurse administrators (*n* = 12) Preceptors (*n* = 66) New graduates (*n* = 43)	Baptist Health Lexington, Baptist Health Madisonville, Hardin Memorial Hospital, and Baptist Health Corbin	Baptist Health Lexington Preceptor PPA has strong content validity, consistency over time, and internal consistency		Baptist Health Lexington PPA	Assessment Tool Validity and Reliability

Panzavecchia and Pearce 2014	Ascertained support provided to preceptors and qualities required to support newly qualified professionals	Preceptors (*n* = 30)	Three hospital sites within one acute trust	Findings useful for planning and developing preceptorship programs; sustainable support for preceptor and preceptee development; lack of role preparation, expectations, and perceived limitations and difficulties	Colaizzi's methodological framework (Sanders, 2003)		Program Planning and Sustainable Support

Hsu et al. 2014	Investigated teaching competencies required in modern, student-centered higher education teaching contexts	Clinical nursing preceptors (*n* = 389)	One regional hospital and two medical centers in northern Taiwan	Clinical Teaching Competence Inventory has adequate construct validity and internal consistency for clinical nursing preceptors to assess clinical teaching behaviors in practice settings	Sonthisombat's model (Sonthisombat 2008)	Clinical Teaching Competencies Scale, Cronbach's *α* = 0.82–0.87	Clinical Teaching Competence Assessment

Hansen 2021	Assessed newly qualified nurses' perceptions of preceptor guidance towards becoming experts in practice at Level II regional hospital in Western Cape	*n* = 162; RNs (48.2%), enrolled nurses (32.7%), enrolled nursing auxiliaries (19.1%)	A selected Level II regional hospital in the Western Cape	Preceptorship supports newly qualified nurses by easing transition from student to practicing nurse and reducing theory-practice gap; ongoing support programs essential after new qualification or/and becoming new nurse	Benner's Novice to Expert model (Benner 1982)		Transition Support and Theory-Practice Integration

L'Ecuyer 2019	Described nurse preceptors' perceptions of nursing students and new graduate nurses with learning disabilities in clinical settings	Preceptors (*n* = 166)	A workshop sponsored by the Missouri Hospital Association	Preceptors felt unprepared and lacked confidence in role for those with learning disabilities; educational modules and support needed in preceptor training to increase preparedness and confidence	Goffman's (1963) stigma theory	A 23-question survey	Preparedness and Confidence for Special Needs Learners

Martínez-Linares et al. 2019	Understood fourth-year nursing students' and Newly Qualified Nurses' perception of lecturers' and clinical preceptors' effectiveness	Newly qualified nurses (*n* = 12) and fourth-year students of the degree (*n* = 12)	A Spanish University	Emphasized need for mentorship training programs; educators worldwide should be appropriately qualified to train future qualified caregivers			Qualified Mentorship Training Delivery

Quek et al. 2019	Explored perceptions, experiences, and needs of nursing preceptors and preceptees on preceptorship	Preceptor-preceptee pairs (*n* = 10)	An acute tertiary hospital in Singapore	Preceptorship programs should cater to local nursing population needs; preceptees may have multiple preceptors to expose to different working styles and relieve preceptor stress			Localized Preceptorship Program Design and Multi-Preceptor Use

Moore and Cagle 2012	Assessing new nurses' experience helps identify and address factors to improve internship and residency programs; increases workplace satisfaction and retention	New graduate nurse (*n* = 7)	Three different hospitals within a large health care system in the South-western United States	Preceptor behaviors crucial for new nurses' acquisition of critical nursing skills through “doing, knowing, and thinking”; positive preceptor behaviors increase new nurse satisfaction; evidence-based preceptor and mentoring programs improve nurse retention and support cost-effective, quality patient care	Diekelmann and Diekelmann's (2009) concernful practices (CPs) of “schooling learning teaching”		Positive Preceptor Behaviors Supporting Skill Acquisition and Satisfaction

Alhassan et al. 2022	Explored preceptors' perceptions of support received in preceptorship role, commitment to role, and important incentives	Preceptors (*n* = 154)	Four hospitals in the northern part of Ghana	Preceptors identified preceptor training, continuing education, and textbooks on effective preceptorship as top incentives; many preceptors in		1.Preceptors' perception of support scale (PPS), Cronbach's *α* = 0.767. 2. Preceptor rewards and incentives questionnaire (PRIQ) 3. Commitment to the preceptor role scale (CPR), Cronbach's *α* = 0.809	Incentive Recognition via Training and Resources

Gholizadeh et al. 2022	Assessed preceptor nurses' perceived benefits, rewards, support, and commitment in new nurse preceptorship program in Iran; examined relationships between these concepts	Preceptors (*n* = 45)	Tabriz Children's Hospital	Commitment to preceptor role associated with perceived benefits, rewards, and support; benefits, rewards, recognition, and support integral to optimizing preceptorship program effectiveness	Kanter's theory of structural empowerment	1. Preceptor's Perception of Benefits and Rewards Scale, Cronbach's alphas of 0.75. 2. Preceptor's Perception of Support Scale, Cronbach's alphas of 0.71. 3. Commitment to the Preceptor Role Scale, Cronbach's alphas of 0.88	Role Commitment through Rewards and Support

Macey, Green, and Jarden 2021	Explored ICU nurse preceptors' perceptions of benefits/rewards, supports, and commitment to preceptor role; identified unique experience in Intensive Care Unit (ICU) context	Preceptors (*n* = 33)	A metropolitan hospital ICU located in Victoria, Australia	Commitment to preceptor role increased by highlighting organizational benefits, increasing contact consistency between preceptorship dyads, and increasing access to supports and preparation	Kanter's (1977) model “Structural Determinants of Behaviour in Organizations”	1. Commitment to the Preceptor Role scale (CPR), Cronbach's *α* = 0.80 2. Preceptors' Perceptions of Benefits and Rewards scale (PPBR), Cronbach's *α* = 0.92. 3. Preceptors Perceptions of Supports scale (PPS), Cronbach's *α* = 0.97	Organizational Benefits Awareness and Access to Support

Omer, Suliman, and Moola 2016	Compared similarities and differences in perceptions of roles and responsibilities between nurse preceptors and preceptees; examined importance and frequency of role attendance	Preceptee (*n* = 87) and preceptors (*n* = 62)		Both groups perceived roles and responsibilities as important; significant difference in rating preceptors' frequency of attendance as educators and facilitators		A self-administered questionnaire using Boyer's (2008) roles and responsibilities, Cronbach's *α* = 0.944	Clear Role Responsibilities and Consistent Educator Attendance

Chang et al. 2015	Explored nurse preceptors' perceptions of preceptor training courses; obtained information on their experiences as preceptors	Nurse preceptors (*n* = 386)	Eight hospitals in northern Taiwan	Most necessary course: communication skills; least useful: adult learning theory and principles; current preceptor training courses impractical; content must be altered to fulfill training needs		Training course perception scale	Communication Skills Training and Content Adaptation

Cloete and Jeggels 2014	Explored nurse preceptors' perceptions of benefits, support, and commitment to preceptor role	Preceptors (*n* = 60)	University of the Western Cape	Nurse preceptors experienced benefits, rewards, and support for preceptorship; committed to their role	Kanter's model of structural determinants of behavior in organizations	A questionnaire developed by Dibert and Goldenberg (1995)	Experiencing Benefits and Support for Sustained Role Commitment

*Note:* Blank space indicates that the content is not mentioned in the article.

Abbreviations: CNS, Clinical Nurse Specialist; CRNA, Certified Registered Nurse Anesthetist; MSPM, Mentorship Support Program Model; NGRN, Newly Graduated Registered Nurse; NHS, National Health Service; RNs, Registered Nurses.

**Table 5:** Nurse preceptor competencies framework.

Category	Competency description	Explanation/examples
Role model	✓ Embody professional and personal attributes such as self-sacrifice, commitment, and leadership ✓ Serve as examples for new nurses, transferring values and behaviors ✓ Uphold continuous competence improvement	Nurse preceptors are expected to lead by example, demonstrating professional nursing behaviors and ethical standards, influencing new nurses positively through their actions and attitudes. Commitment to the role and leadership facilitates safe and positive practice transition

Teaching Competency	✓ Pedagogical skills including teaching ability, clinical competence, evaluation, communication, feedback provision ✓ Structured training and education preparation before and during preceptorship ✓ Ability to facilitate clinical judgment and critical thinking ✓ Use of evidence-based, competency-based curricula and assessment tools ✓ Flexibility to cater to diverse learner needs and clinical contexts	Teaching competence is fundamental, comprising both nursing and educational skills. Preceptors need preparation programs, training models, tools to measure teaching and evaluation, and the ability to provide constructive and timely feedback. They develop critical reasoning and facilitate clinical judgment for learners

Motivation Competency	✓ Commitment and dedication towards preceptorship role and future workforce development ✓ Perceived benefits, rewards, recognition, and support increase motivation ✓ Need for organizational and peer support ✓ Motivation influenced by workload management and clear role expectations	Motivation competency refers to the preceptor's dedication and willingness to teach, fostered by internal factors (recognition, satisfaction) and external supports (manager, colleague, organizational resources). Managing workload and clear expectations improve retention and motivation to perform effectively

Personal Attribute Competency	✓ Interpersonal relationships, communication skills, empathy, cultural competence ✓ Adaptability and willingness to learn and improve ✓ Patience and the ability to manage challenges ✓ Reflective practice (as a skill) and critical reasoning disposition	Attributes and skills contributing to successful preceptoring relationships include communication skills, emotional intelligence, cultural competence, and reflective practice. These aid in supporting diverse learners, navigating stress, and fostering professional growth for both preceptors and preceptees

*Note:* Competencies were derived from thematic synthesis of 48 included studies.

## Data Availability

The data used in this study are obtained from systematic searches conducted in four databases: PubMed, Embase, Web of Science, and Cochrane Library. The search period ranged from January 2012 to September 2022. All search records and selected literature data can be made available upon request to the corresponding author.
